# Editorial—Our Aim

**Published:** 1893-11

**Authors:** 


### EDITOR'S OUTLOOK.

### OUR AIM.

Hall's Journal of Health is designed to answer the precise purpose indicated by its title—to supply a fund of instruction in regard to the laws of the human body, which is nowhere else to be found in popular form. Some of our readers appear to have imbibed the idea, that we hang all our hopes of promoting human health and longevity on a mere reformation in eating and drinking.

We are not disposed to make everything of eating and drinking; their connection with human health is indeed obvious and important; but so is that of air, temperature, dress, and the state of mind, affections and passions.

Health is an article of manufacture, and not a gift of Providence, in the sense commonly received.

True, it is a gift of God, just as skill in music or a profound knowledge of mathematics is a gift, *and in no other way.* We do not doubt that some persons inherit a predisposition to certain diseases,—the iniquities of the fathers being thus often visited upon the children unto the third and fourth generation,—still, by obedience to the laws of life, every individual can just as surely improve health—*manufacture it,* even—as improve his mind or heart.

In short, the idea prevails that health is an article of human manufacture, as much so as any mental or moral acquirement—and the world is, prospectively reformed.

In our December issue, we inaugurate our Winter Recreation Bureau and respectfully invite all Health resort hotels to send us their circulars. December first, we will be able to give any information desired of any winter health resort in the world.

